# In Vitro and In Vivo Antiviral Activity of a Modified Medium-Chain Fatty Acid Against Porcine Reproductive and Respiratory Syndrome Virus

**DOI:** 10.3390/pathogens15080823

**Published:** 2026-08-05

**Authors:** Nader Maher Sobhy, Valeria Lugo-Mesa, Christiana Rezk Bottros Youssef, Cecilia Balestreri, Yuan-Tai Hung, Gary D. Reznik, Declan C. Schroeder, Sagar Mal Goyal

**Affiliations:** 1 Department of Veterinary Population Medicine, University of Minnesota, St. Paul, MN 55108, USA; nyaacoob@umn.edu (N.M.S.); valerialugo@tamu.edu (V.L.-M.); cyoussef@umn.edu (C.R.B.Y.); cecilia.balestreri@gmail.com (C.B.); dcschroe@umn.edu (D.C.S.); goyal001@umn.edu (S.M.G.); 2 Department of Animal Medicine, Faculty of Veterinary Medicine, Zagazig University, Zagazig 44511, Sharkia, Egypt; 3 Department of Large Animal Clinical Sciences, College of Veterinary Medicine and Biomedical Sciences, Texas A&M University, Canyon, TX 79015, USA; 4 Department of Microbiology and Immunology, Faculty of Pharmacy, Zagazig University, Zagazig 44519, Sharkia, Egypt; 5 Devenish Nutrition LLC, 2320 Lake Ave., Fairmont, MN 56031, USA; yuantai.anivet@gmail.com

**Keywords:** pigs, mMCFA, PRRSV, D-Strike, feed efficiency, viability RT-PCR

## Abstract

Porcine reproductive and respiratory syndrome virus (PRRSV) causes significant economic losses in the global swine industry, partly because current vaccination strategies offer limited protection against heterologous viral strains. Since modified medium-chain fatty acids (mMCFA) offer enhanced structural stability and antimicrobial properties compared with unmodified MCFA, we evaluated a proprietary mMCFA blend (D-Strike, Devenish Nutrition) against PRRSV in vitro and in vivo. In vitro efficacy was assessed using TCID_50_ assays on MARC-145 cells and a viability qPCR (V-qPCR). At 1:1000, 1:2000, and 1:5000 dilutions, the mMCFA inactivated >4 logs (99.99%) of the virus within 5 min of exposure. The results of V-qPCR were also compatible with those obtained by cell culture assays. For the in vivo study, 40 weaned male piglets were assigned to four dietary treatments: negative control (group A), PRRSV-challenge control (group B), 0.2% D-Strike (group C), and 0.4% D-Strike (group D). Pigs in groups B, C, and D were challenged intranasally with virulent PRRSV. The results demonstrated that dietary mMCFA reduced clinical signs, attenuated a febrile response, and decreased the severity of lung lesions. Some responses appeared more pronounced at the 0.4% inclusion level than at 0.2%, although a formal dose–response analysis was not statistically significant for most outcome measures. The sera from the 0.4% D-Strike group showed 65% lower PRRSV than the positive control, although this difference was not statistically significant. In addition, this group had 0% mortality as compared to 20% in the positive control group. Feed efficiency also improved in D-Strike-fed pigs. These findings indicate that mMCFA shows promise as a feed additive for mitigating PRRSV infection, although the in vivo antiviral effects require confirmation in larger studies.

## 1. Introduction

Porcine reproductive and respiratory syndrome (PRRS), caused by PRRS virus (PRRSV), is a disastrous disease with severe economic impacts on the pig industry. Losses from PRRS are attributed to a reduction in reproductive performance, feed efficiency, and weight gain, as well as increases in treatment costs, morbidity, and mortality [1]. PRRSV is an enveloped, single-stranded RNA virus belonging to the genus *Betaarterivirus* in the family *Arteriviridae*. There are two genotypes: PRRSV-1 and PRRSV-2, representing European (prototype Lelystad) and North American (VR-2332) genotypes, respectively [2].

A high frequency of mutation and/or recombination is responsible for continued outbreaks of this disease worldwide [3,4]. The virus can lead to persistent infections in swine herds and facilitate infection with secondary viral and bacterial infections [5,6]. It is important, therefore, to evaluate technologies that can mitigate pathogenic challenges and reduce viral transmission among herds.

Medium-chain fatty acids (MCFA) consist of 6–12 carbon atoms. They are mainly derived from milk, coconut oil, and palm kernel oil [7]. For example, lauric acid constitutes about 53% of fatty acids in coconut oil, shows no cytotoxicity to rat lymphocytes, and has been approved for food usage by several organizations, including the U.S. Food and Drug Administration and the European Union [8]. The digestion and absorption of MCFA is more efficient than long-chain triglycerides; thus, pigs fed diets containing MCFA have shown improvements in growth performance, reproductive efficiency, and gastrointestinal health [9]. In addition, MCFA can mitigate infections with *Streptococcus suis*, *coccidia*, *Escherichia coli*, *Clostridium* spp., and *Salmonella* spp. [10]. MCFA supplementation can also help to reduce the use of antibiotics in farm animal production, thereby reducing the development of antimicrobial resistance in bacterial pathogens [11].

Recently, MCFA, in the form of free fatty acids, was found to have antiviral activities against porcine epidemic diarrhea virus, African swine fever virus, and Seneca virus A [12,13,14,15]. Unfortunately, the in vivo efficacy of non-modified MCFA is lost or limited due to chemical and enzymatic hydrolysis in the body. Recently, modified MCFA (mMCFA) have been developed, which are more structurally stable than non-modified MCFAs. Thus, mMCFA represents a novel approach that can support animals during pathogenic challenges with Gram-positive bacteria and enveloped viruses. We hypothesized that a proprietary blend of mMCFA may reduce PRRSV activity. Hence, the objective of this study was to examine the activity of a proprietary blend of mMCFA against in vitro and in vivo PRRSV in piglets.

## 2. Materials and Methods

### 2.1. Cells and Virus

PRRSV-2 (strain 1-4-4 MN) was propagated and titrated on MARC-145 cells, which are a subclone of African green monkey kidney-derived MA-104 cells [16]. The virus was originally isolated from a clinical outbreak in Minnesota, then sequenced and deposited in GenBank under accession number MZ423535.1. The cells were grown in modified Eagle’s medium (MEM) (Corning, Glendale, AZ, USA) with 4% fetal bovine serum (Cytiva, Marlborough, MA, USA), 50 µg/mL of neomycin (Sigma-Aldrich, St. Louis, MO, USA), 1 µg/mL of fungizone (Sigma-Aldrich, St. Louis, MO, USA), 150 µg/mL of streptomycin (Sigma-Aldrich, St. Louis, MO, USA), and 150 IU/mL of penicillin G potassium (Sigma-Aldrich, St. Louis, MO, USA). The virus was harvested after 80% of the cells showed cytopathic effects. Aliquots of the stock virus were stored at −80 °C until used in the experiments.

### 2.2. Viability qPCR (V-qPCR)

This method is described in Balestreri et al. (2024) [17]. PRRSV was mixed with different dilutions of mMCFA (1:1000, 1:2000, and 1:5000) and incubated at room temperature for different time points. Thereafter, these dilutions were treated with PMAxx dye (100 µM final concentration, Biotium Inc, Fremont, Canada) and incubated in the dark at room temperature for 10 min on a rocker for optimal mixing. The mixed sample was then exposed to light for 30 min using a PMA-Lite device (Biotium Inc, Fremont, QC, Canada) to cross-link the PMAxx dye to free RNA. RNA was extracted using the NucleoMag^®^ VET (Takara Bio, San Jose, CA, USA) kit, and the RT-qPCR was performed using the QuantiNova SYBR Green RT-PCR kit (Qiagen, Toronto, ON, Canada) under the conditions described by Balestreri et al. (2024) [17]. Standard curves for the RT-qPCR assays were generated by making 10-fold serial dilutions of PRRSV at a known titer (TCID_50_). All RT-qPCR assays were conducted using a Roto-gene Q Real-Time PCR (Qiagen, Toronto, ON, Canada).

### 2.3. In Vitro Experiment

A water-soluble proprietary blend of mMCFA (D-Strike, Devenish Nutrition, Fairmont, MN, USA) was used. D-Strike is a feed-grade lactylated fatty acid-based anionic surfactant. Four dilutions (1:1000, 1:2000, 1:5000, and 1:10,000) of mMCFA were prepared in MEM. An equal volume (0.5 mL) of PRRSV was added to 0.5 mL of each dilution, resulting in final dilutions of 1:2000, 1:4000, 1:10,000, and 1:20,000. Samples were collected from each tube after 1, 2, and 5 min of incubation at room temperature. Serial 10-fold dilutions of these samples were prepared in MEM, followed by inoculation onto monolayers of MARC-145 cells in 96-well plates using 3 wells per dilution. The inoculated plates were incubated at 37 °C for 7 days. The plates were examined daily under an inverted microscope for the appearance of virus-induced cytopathic effects. Virus titers were calculated according to the Karber–Spearman method [18].

Untreated virus controls (virus in MEM without mMCFA) were included in each experiment to establish baseline infectivity. All experiments were performed in triplicate (three independent biological replicates), with three technical replicates per dilution. To confirm that the antiviral effects were not secondary to cellular toxicity, MARC-145 cell viability was assessed following exposure to mMCFA. Cells were treated with mMCFA dilutions of 1:100, 1:200, 1:500, 1:1000, and 1:2000 (final dilutions after mixing with an equal volume of cell culture medium) and incubated for 24 and 48 h. Cell viability was evaluated using the trypan blue exclusion assay. The 1:1000 final dilution was found to be toxic to MARC-145 cells, with viability reduced to <70% at 48 h. Therefore, all antiviral experiments were conducted using mMCFA dilutions that did not affect cell viability (1:2000, 1:4000, 1:10,000, and 1:20,000 final dilutions, corresponding to initial dilutions of 1:1000, 1:2000, 1:5000, and 1:10,000, respectively), ensuring that the observed antiviral effects were not attributable to cytotoxicity.

### 2.4. In Vivo Experiment

After demonstrating the in vitro efficacy of mMCFA against PRRSV, a study was conducted in pigs to determine whether mMCFA could protect against PRRSV infection. However, the actual adverse effects on animals may be different because of the interaction of many physiological factors that are absent in the in vitro assay [19].

#### 2.4.1. Preparation and Administration of mMCFA

Two inclusion rates of mMCFA (0.2% and 0.4%) in single-phase nursery diets replacing corn were examined. A total of 4 different feed types (400 lbs each) were supplied by Devenish Nutrition, e.g., a basal nursery diet for the negative control group, a basal nursery diet for the positive control group, a basal nursery diet plus 0.2% mMCFA for the low-dose group, and a basal nursery diet plus 0.4% mMCFA for the high-dose group. Diets did not contain specialty additives but did contain super-dose phytase at 2000 FTU/kg, pharmacological Zn at 3000 ppm, and elevated Cu at 199 ppm. On receipt, samples of feed were tested by rRT-PCR and were found to be negative for PRRSV.

#### 2.4.2. Animals and Groups

The trial was conducted at the University of Minnesota Veterinary Isolation Facility, St. Paul, MN, USA. A total of 40 recently weaned male piglets of approximately 21 days of age were randomly allotted to four different dietary treatments in 4 different isolators. Upon arrival, three pigs developed notable diarrhea and were excluded from the study before treatment allocation. The remaining 37 pigs were randomly assigned to groups as follows: group A (*n* = 9), group B (*n* = 10), group C (*n* = 10), and group D (*n* = 8). The unequal group sizes resulted from the exclusion of the three pigs with diarrhea and the subsequent allocation of the remaining pigs to maintain balance as much as possible. Pigs had free access to a feeder per room, and feed pan coverage was targeted at about 75%. Pigs in groups B, C, and D were challenged intranasally with virulent PRRSV (Lineage 1C 1-4-4; 1 mL/nostril/pig) on day 8. The virus titer was 10^5^ TCID_50_/mL. The experimental design is summarized in Table 1 and Table 2. The amount of feed added to the feeder during the trial was recorded and weighed at the end of the trial to calculate the average feed consumption for each treatment.

#### 2.4.3. Clinical Signs

Clinical signs, including fever and cough, were monitored daily by trained observers who were blinded to treatment allocation. The observers were unaware of which diet each group had received. Body temperature was recorded daily post-infection (Days 9–17 in Table 1) through a microchip (Merck Bio-Thermo chips, Rahway, NJ, USA) installed in each animal’s ear. An accompanying thermal scanner provided data on the body temperature of pigs without the need to handle them. All pigs were weighed before recruitment in the study and daily after challenge until euthanasia.

#### 2.4.4. Sample Collection and Testing

The animals were bled on days 11 and 18 (3 and 10 days post-infection). Sera from these blood samples were tested for viral nucleic acid by rRT-PCR. All pigs were euthanized on day 18 (10 days post-infection, Table 2) with approval from the University of Minnesota’s IACUC. Serum samples from euthanized pigs were stored in a −80 °C freezer until tested by RT-PCR. After euthanasia, necropsy was performed, lung specimens from all pigs were collected and fixed in 10% buffered formalin, followed by histopathological examination according to Slaoui (2011) [20]. Slides were coded and examined by a veterinary pathologist who was blinded to treatment allocation. Lesions were categorized as None, Mild, Moderate, or Severe based on predetermined criteria.

To avoid potential bias associated with the industry affiliations of some co-authors, the following measures were implemented: (1) clinical signs were assessed by trained observers who were blinded to treatment allocation; (2) histological evaluation was performed by a veterinary pathologist blinded to treatment allocation; (3) statistical analyses were performed by investigators who were not directly involved in the conduct of the in vivo study; and (4) feed allocation and preparation were performed by personnel not involved in animal observations or outcome assessments. The industry-affiliated co-authors (Y.-T.H. and G.D.R.) participated in the study design and data interpretation but were not involved in daily clinical observations, sample collection, or histological scoring so as to minimize potential bias.

#### 2.4.5. Statistical Analysis

All statistical analyses were performed using SAS version 9.4 (SAS Institute, Cary, NC, USA). For in vitro data, virus titers between treatment groups were compared using one-way ANOVA with Tukey’s post hoc test. For in vivo data, the growth performance data were analyzed using a completely randomized design with linear mixed models with repeated effects. Treatment means were separated using the PDIFF option with the Tukey–Kramer adjustment for multiple comparisons. For the variable “temperature,” data were analyzed using the PROC FREQ procedure in SAS. If the chi-square was significant, then post hoc tests for multiple comparisons were performed. For the variable “virus particles,” data were analyzed using the Kruskal–Wallis test in a nonparametric one-way ANOVA. Significance is defined at *p* < 0.05, while a tendency is considered when 0.05 < *p* ≤ 0.10. All data were tested for normality using the Shapiro–Wilk test before parametric analyses.

## 3. Results

### 3.1. In Vitro Study

As far as virus inactivation is concerned, the tested dilutions of D-Strike (1:1000, 1:2000, and 1: 5000) were very potent. These dilutions were able to kill >2 logs of PRRSV almost instantaneously. At a 1:1000 initial dilution (final dilution 1:2000), this formula inactivated 99.95% and 99.97% of the virus within 1 min and 2 min, respectively (Table 3). At a 1:2000 dilution, 99.78% and 99.97% of the virus were inactivated within 1 min and 2 min, respectively. D-Strike at initial dilutions from 1:1000 to 1:5000 killed > 4 logs or 99.99% of PRRSV in the 5 min exposure. However, the 1:10,000 dilution was not very effective since it inactivated only 53% of the virus after 5 min (Table 3).

**Figure 1:**
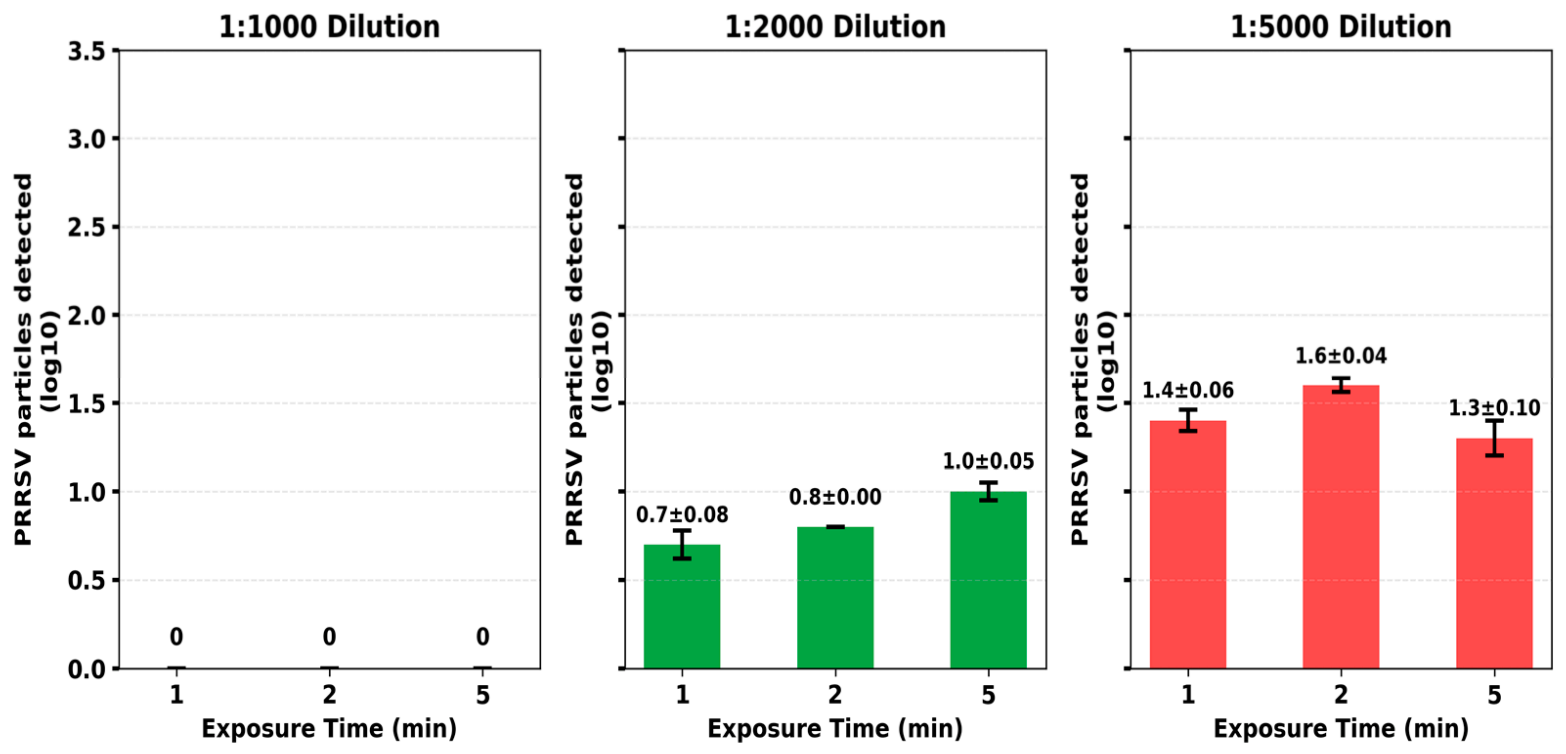
Viability qPCR results for the three dilutions (1:1000, 1:2000, and 1:5000) over three time exposures (1, 2, and 5 min). The starting quantity of virus was at 3.5 log 10 on the Y-axis. The absence of signals in the 1:1000 dilution treated samples indicates complete elimination of the treated virus.

Three dilutions of mMCFA (1:1000, 1:2000, and 1:5000) plus time (1, 2, and 5 min) exposure treatments were further tested using the V-qPCR assay to determine whether virus particles remained intact after treatment. Every increase of every three values in CT was considered as one log reduction [17]. An average of ~1.5 log reduction was observed for the 1:5000 dilution, ~2 log reduction for the 1:2000 dilution, and 100% kill for the 1:1000 dilution (Figure 1). Time had little or no effect, i.e., the mMCFA treatment was equally effective after 1, 2, and 5 min exposures. All in vitro experiments were performed in triplicate. Virus titers were calculated according to the Karber–Spearman method. Percent virus inactivation was calculated as (A − B/A) × 100, where A = initial virus titer and B = remaining virus titer after mMCFA exposure. The 1:1000 to 1:5000 dilutions consistently reduced virus titers by >4 logs across all three independent experiments, with no variability between replicates.

### 3.2. In Vivo Study

Three pigs died due to notable diarrhea upon arrival and hence were excluded from the study. Of the remaining 37 pigs, three died 1–2 days before euthanasia. The carcasses of these three pigs were refrigerated and subjected to necropsy and histopathology, along with the other 34 pigs that were euthanized on day 18.

#### 3.2.1. Clinical Assessment

**Figure 2:**
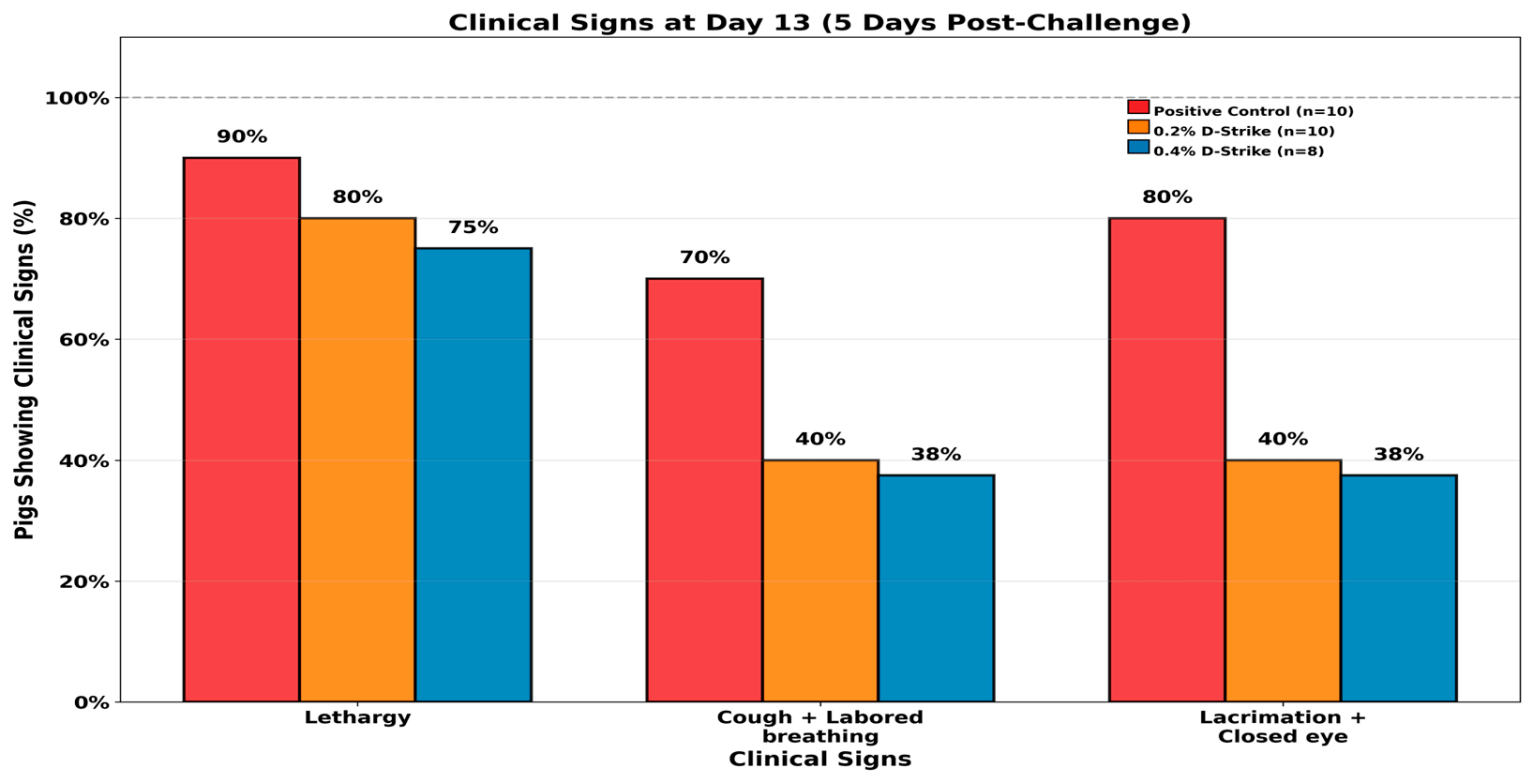
Clinical signs observed on day 13 (5 days post-challenge) across treatment groups. Data are presented as the percentage of pigs showing each clinical sign (count/*n* × 100). Positive control group (Group B, *n* = 10). Low-dose D-Strike group (Group C, *n* = 10). High-dose D-Strike group (Group D, *n* = 8).

Clinical signs were observed daily until the end of the study. No clinical signs were seen in the negative control group (group A) during the study. Pigs in groups B, C, and D showed varying degrees of clinical signs, e.g., lethargy, coughing, labored breathing, closed eyes, and lacrimation (Figure 2). The descriptive data suggests a reduction in clinical signs in D-Strike-fed pigs; however, due to the qualitative nature of clinical scoring and small group sizes, formal statistical analysis was not performed on these observations.

#### 3.2.2. Body Temperature

**Figure 3:**
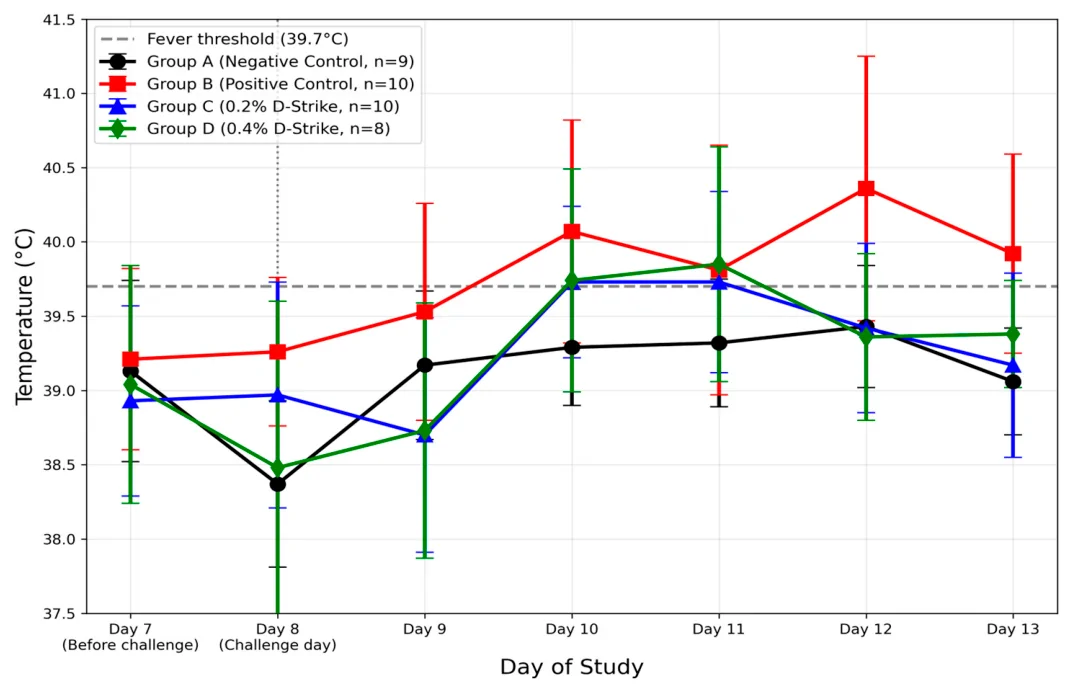
Mean SD of temperature up to 5 days post-inoculation of pigs infected with PRRSV. Individual pig temperatures are shown as colored dashes, with group means represented by dense geometric shapes.

**Figure 4:**
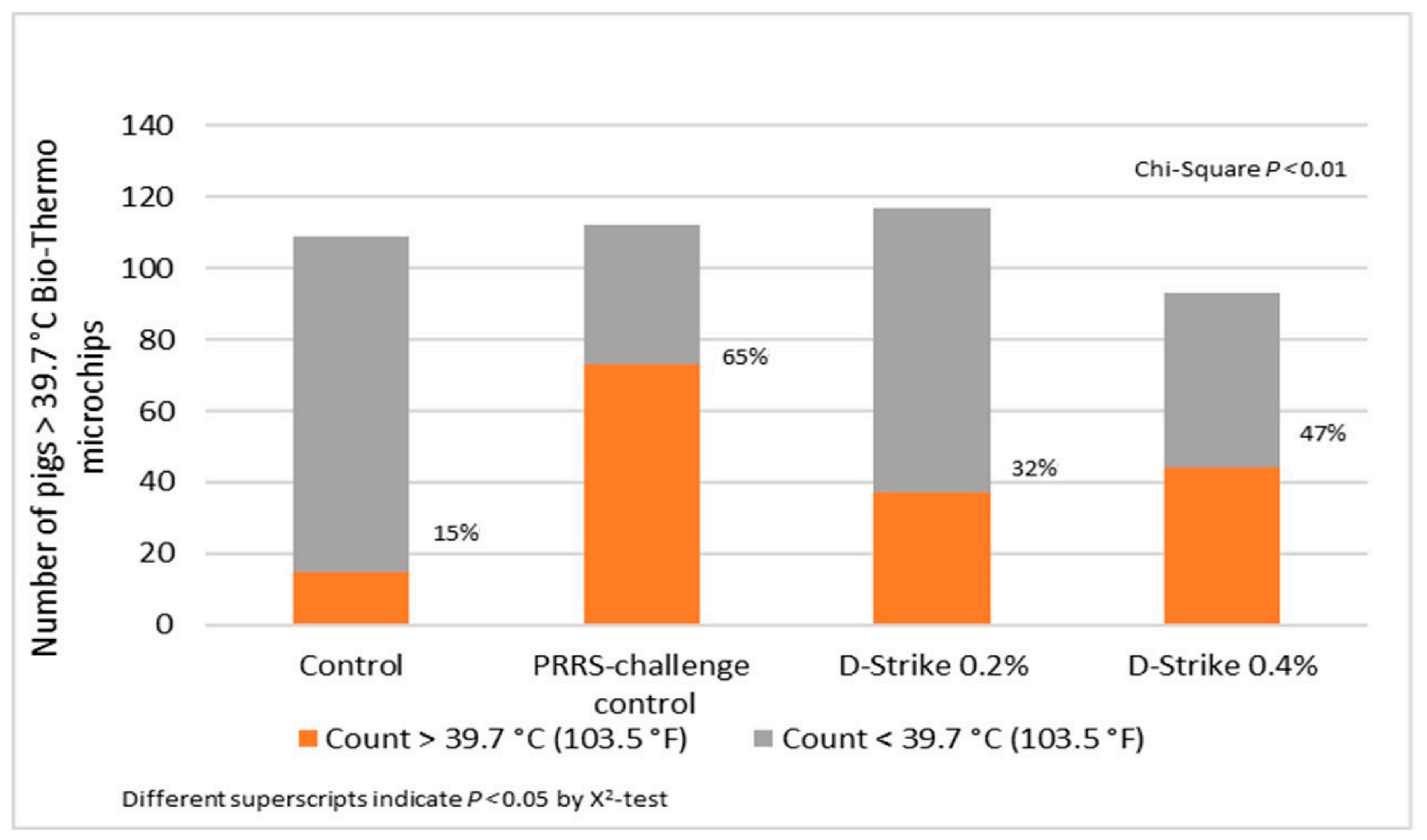
Counts of pigs with fever from Bio-Thermo microchips from a day before challenge to the day of euthanasia.

Temperatures were recorded daily, but for statistical purposes, only those taken on the days of arrival, challenge, and euthanasia were analyzed (Figure 3 and Figure 4). Statistical analysis was done by repeated-measures ANOVA, and normality was tested by the Shapiro–Wilk test. The temperature of the positive control group (group B) reached 40 ± 0.3 on the second day after challenge, then fluctuated +/−0.1–0.7 in all animals of that group up to the day of euthanasia (10 days after challenge). The treated group C temperature reached 40 +/−0.1–0.7 in half of the group (5/10) and then fluctuated +/−0.5–1 in all animals of that group up to the day of euthanasia. The treated group D temperatures reached 40 +/−0.1–0.4 in five of eight pigs and then fluctuated +/−0.2–0.3 in all animals of that group up to the day of euthanasia. The data were normally distributed, and mMCFA showed a significant delay in raising the temperatures in D-Strike groups at *p* < 0.05.

#### 3.2.3. Mortality

Three pigs died 1–2 days before euthanasia. The carcasses of these three pigs were refrigerated and subjected to necropsy and histopathology, along with the other 34 pigs that were euthanized on day 18. Mortality was 20% (2/10) in the positive control group, 10% (1/10) in the 0.2% D-Strike group, and 0% (0/8) in the 0.4% D-Strike group. No mortality occurred in the negative control group. While the descriptive data suggest a dose-dependent reduction in mortality, the small sample size and low event rate preclude definitive statistical conclusions.

#### 3.2.4. Histopathology

**Figure 5:**
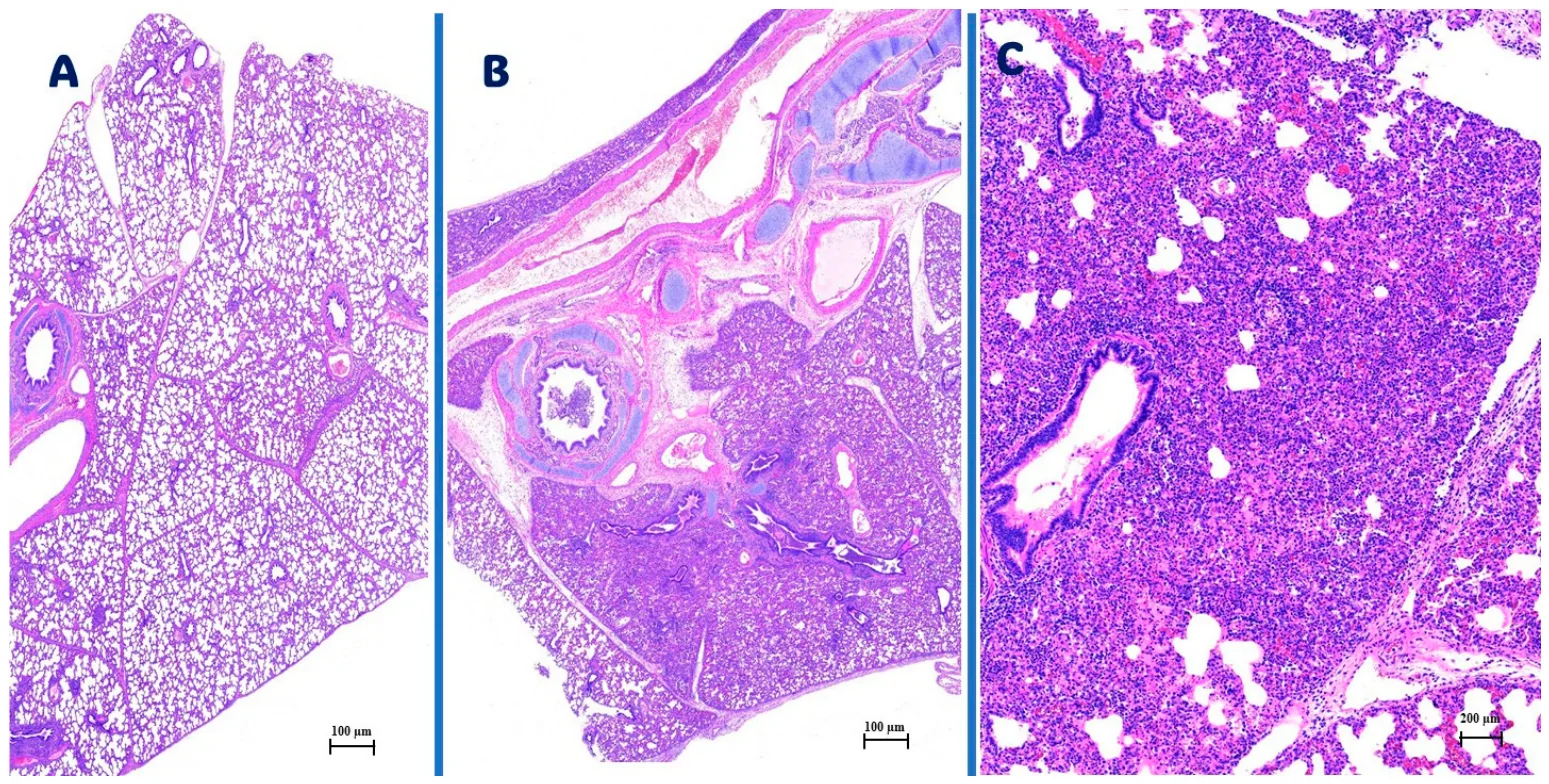
Representative histopathological images of lung tissue sections (H&E staining, 10× for (**A**,**B**) and 20× magnification for (**C**)). (**A**) Negative control: normal lung architecture. (**B**) High-dose D-Strike (0.4%): reduced lesion severity with moderate septal thickening and decreased inflammation. Lesion severity scoring revealed numerical trends. Positive control: severe interstitial pneumonia with marked alveolar septal thickening and inflammatory infiltration (**C**).

Lung lesions were examined in sections from the mid-apical lobe, mid-middle lobe, and dorsal caudal lobe. Lesions were categorized as follows: None = no lung lesion, Mild = slight congestion in one, two, or three sections, Moderate = one or two of the three sections showing severe lesions, and Severe = three of three sections showing severe lesions. Representative images for lung lesions are shown in Figure 5. No histological lesions were seen in the negative control group. All pigs in the PRRSV-challenge control group (group B) had severe bronchopneumonia and interstitial pneumonia. In contrast, groups fed D-Strike had less severe lesions compared to the PRRSV-challenge control pigs, including fewer necrotic macrophage debris (Table 4). The proportion of pigs with severe lung lesions was 87.5% in the positive control group, 77.8% in the 0.2% D-Strike group, and 50% in the 0.4% D-Strike group. While this pattern suggests a possible dose-dependent effect, statistical testing was not performed due to the categorical nature of the data and small sample sizes.

#### 3.2.5. Detection of PRRSV RNA in Serum Samples

**Figure 6:**
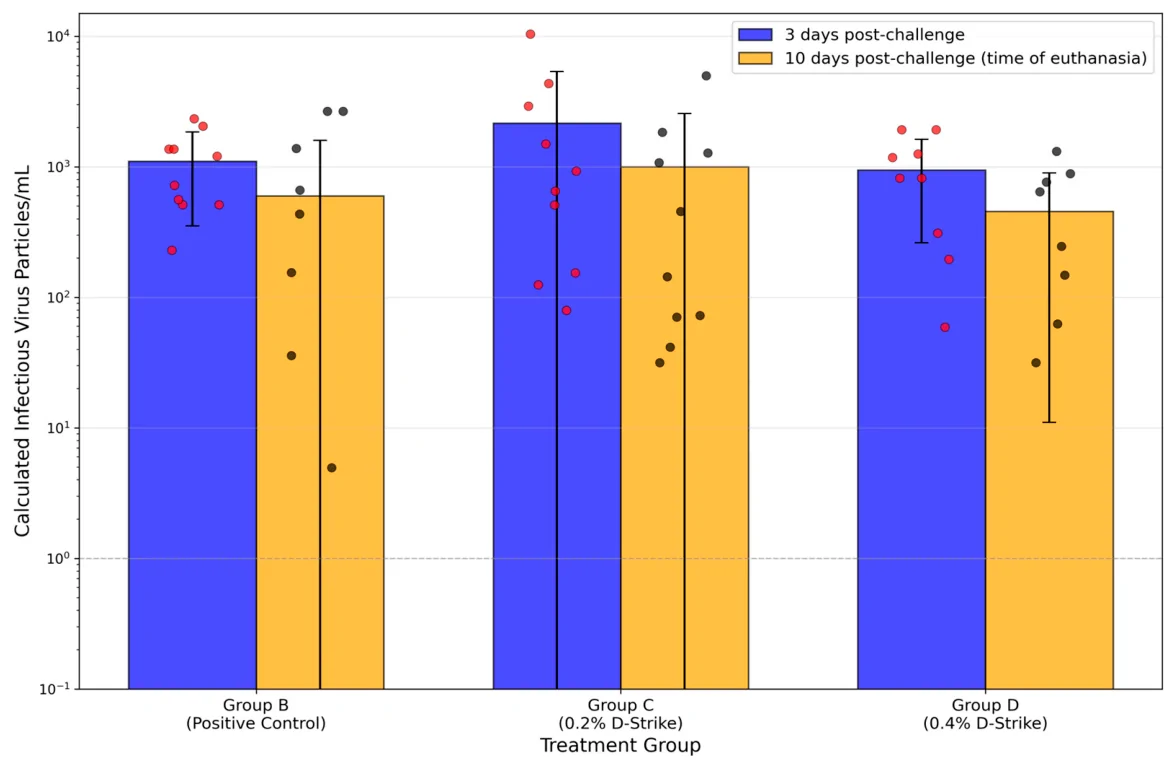
Comparison of calculated infectious PRRSV particles/mL in serum at 3 days post-challenge (blue bars, red scatter dots) and 10 days post-challenge (orange bars, black scatter dots) for Groups B (positive control, *n* = 10), C (0.2% D-Strike, *n* = 10), and D (0.4% D-Strike, *n* = 9). Data are presented as means ± standard deviations. Individual animal data points are shown as scatter dots (red = 3 dpi, black = 10 dpi) to illustrate the substantial biological variability within each treatment group. The y-axis is presented on a logarithmic scale (10^x^ notation) to accommodate the wide range of viral loads observed.

All serum samples collected on the third day of arrival were negative for PRRSV when tested by qRT-PCR. Serum samples collected on day 11 (third day post-challenge) and day 18 (day of euthanasia) were also examined by qRT-PCR. No PRRSV was detected in the negative control group. After the challenge, all pigs showed systemic PRRSV with varying levels between groups. The mean virus particles/mL were 959, 2161, and 1217 in groups fed D-Strike 0.4, D-Strike 0.2, and the positive control group after challenge, and 512, 998, and 1468 in the same groups, respectively, at euthanasia (Figure 6). The mean virus concentration in pigs fed D-Strike at 0.2% and 0.4% was approximately 32% and 65% lower on day 18, respectively, as compared to the positive control group (group B). Although the Kruskal–Wallis test did not indicate any significant differences in serum virus concentrations on day 11 (*p* = 0.72) and day 18 (*p* = 0.88), descriptive statistics revealed that infectious virus particles had increased in the positive control (group B) but were reduced in the sera of D-Strike-fed pigs. These trends, while not statistically significant, suggest a potential antiviral effect that warrants further investigation.

#### 3.2.6. Growth Response

We considered only the initial weight (day 3), weight at challenge (day 8), and weight collected after euthanasia (day 18). Before challenge (day 8), pigs in the non-challenge control had a better growth response, probably due to less stress and diarrhea incidence upon arrival in the other groups. Body weight and gain were depressed sharply (*p* < 0.01; Table 5) compared to the non-challenge control post-infection. Each isolation unit was equipped with one feeder; thus, the mean feed consumption of the pigs in each isolation unit was used to calculate feed efficiency. A descriptive statistic suggests that pigs fed D-Strike at either level had better gain and feed efficiency compared to the PRRSV-challenge control (Table 5). However, it is important to note that these differences were not statistically significant, likely due to the small group sizes and high individual variability in feed intake and weight gain post-challenge. The numerical trends should therefore be interpreted with appropriate caution.

## 4. Discussion

In vitro data show that 1:1000 to 1:5000 dilutions of mMCFA effectively reduced the titer of PRRSV by more than 4 logs. The mechanism of inactivation of an enveloped virus by MCFA is not fully understood, but a few studies have suggested that MCFA might interfere with the maturation of an enveloped virus, leading to inhibition of virus release. Esterification reactions between glycerol and MCFA allow the formation of micelles at lower concentrations of MCFA, which indicates why mMCFA are more biologically potent than free MCFA [21]. Recently, Yang et al. (2022) found that caprylic monoglyceride had strong anti-PRRSV activity in vitro and in vivo [22]. The antiviral effects of mMCFA containing lauric acid observed in this experiment agree with those of Tran et al. (2021), who found that mMCFA had an antiviral effect against African swine fever (ASF) virus [23]. Hornung et al. (1994) reported that lauric acid can inhibit the maturation of vesicular stomatitis virus [24]. The antiviral activity of lauric acid is believed to be due to the destruction of the lipid coat of viruses and bacterial cell membranes by physicochemical processes [25].

The V-qPCR results in this study appear to support this mechanism of virus particle damage, as viral damage was observed at all three mMCFA dilutions, irrespective of the exposure time. An alternative or cooperative mechanism of inactivation could be due to interference with cellular signal transcription and transduction. Multiple mechanisms of action are advantageous because they reduce the chances of the virus developing resistance [26]. Thus, an innovative feed additive, such as mMCFA, has the potential to support pig production by decreasing viral insults. However, it is important to note that the mechanistic interpretations offered in this discussion are presented as hypotheses rather than established mechanisms. Our study was designed primarily to evaluate the antiviral activity of mMCFA and did not include specific mechanistic experiments (e.g., assessment of viral envelope integrity by electron microscopy, evaluation of cytokine profiles, or investigation of cellular signaling pathways). Future studies employing such approaches will be necessary to elucidate the precise mechanisms by which mMCFA exerts its observed effects.

Different fatty acids may have different antiviral effects in vitro and in vivo. Thus, the chain length of monocarboxylic acid is considered crucial for an antiviral effect [24]. Many studies have shown a synergistic effect of a combination of two or more MCFAs. A significant decrease in bacterial counts was observed with a combination of caprylic acid and capric acid as compared to the use of an individual MCFA [27]. Lerner et al. (2020) found that a 1:1:1 mixture of hexanoic acid, octanoic acid, and decanoic acid resulted in a significant reduction in porcine epidemic diarrhea virus in swine feed [12]. Blends of caprylic acid, capric acid, and lauric acid at a 1:1:1 ratio exhibited synergistic activity against ASFV compared with the individual acids [23]. This information supports the use of a blend of mMCFA to ensure antibacterial and antiviral activity. However, further studies are needed to elucidate the exact mechanism of action of mMCFA and its interactions with viruses.

In the in vivo part of this study, we found that PRRSV-associated clinical signs—lethargy, labored breathing, coughing, and lacrimation/closed eyes [28]—were markedly higher in the PRRSV-challenged positive control group than in pigs fed D-Strike at either level (Figure 2). Respiratory distress in PRRSV-infected pigs is directly associated with viral replication in pulmonary alveolar macrophages and the resultant lung pathology [29]. Therefore, the observed clinical improvement is likely to be a direct consequence of reduced pulmonary viral burden and lesion severity in pigs fed D-Strike.

Fever is a characteristic acute-phase response following PRRSV infection, which is driven by pro-inflammatory cytokines released from activated macrophages [30]. In the present study, body temperature was monitored continuously using Bio-Thermo micro ear chips. The temperatures recorded by this device are known to correlate well with rectal temperatures in pigs under experimental conditions [3,31]. Although a post-challenge febrile condition was observed in all PRRSV-inoculated groups (Figure 3), the proportion of pigs with body temperature exceeding 39.7 °C (103.5 °F) was significantly lower in the D-Strike groups as compared to the positive control (Figure 4). This indicates that dietary D-Strike at 0.2% and 0.4% can attenuate the febrile response to PRRSV infection. Furthermore, the reduced fever in D-Strike-treated pigs (a 65% reduction with 0.4% D-Strike) and less severe lung lesions (only 50% severe lesions in the 0.4% group vs. 87.5% in the positive control) indicate the utility of using this mMCFA. Viral load is a major driver of the systemic inflammatory response, and a decrease in viremia is expected to reduce pyrexia [32]. Thus, the antipyretic effect of D-Strike is likely mediated through its antiviral activity rather than a direct antipyretic mechanism. The apparent dose-dependent trend observed for several outcomes (mortality: 20%, 10%, and 0%; severe lung lesions: 87.5%, 77.8%, and 50% for control, 0.2%, and 0.4% D-Strike, respectively) is suggestive of a dose–response relationship. However, the small sample size and descriptive nature of these data preclude definitive conclusions regarding dose-dependency. These findings should be considered preliminary and require confirmation in larger studies designed to detect dose–response effects.

At the end of the in vivo study, 0% mortality was observed in the 0.4% D-Strike group, compared to 20% and 10% mortality, respectively, in the positive control and the 0.2% D-Strike group (Table 4). This dose-dependent protective effect has been shown with other related compounds in previous studies. For instance, piglets treated with higher concentrations of caprylic monoglyceride (CMG) had significantly lower mortality and reduced viral loads after PRRSV infection as compared to the untreated controls [22]. Similarly, feeding 1-α-glycerol monolaurate (GML) at 2 kg/mT resulted in no clinical signs of PRRS or viremia, whereas 100% of positive control pigs exhibited clinical signs and tested positive for the virus [33].

The histopathological assessment further supports the clinical findings. While all pigs in the PRRSV-challenge control group developed severe bronchopneumonia and interstitial pneumonia, D-Strike-fed pigs showed a marked reduction in the severity of the lesions. Specifically, the 0.4% group had only 50% of pigs exhibiting lesions, compared to 87.5% in the positive control group. This improvement in pulmonary pathology is a key indicator of the compound’s efficacy. PRRSV primarily replicates in porcine alveolar macrophages, leading to interstitial pneumonia characterized by thickened alveolar septa and inflammatory cell infiltration [34]. The reduction in severe lesions suggests that D-Strike may either inhibit viral replication within the lungs or modulate the host’s pathological inflammatory response to the virus.

The results were also supported by qRT-PCR. It is well-established that the magnitude of biological responses in pigs undergoing the PRRSV challenge is directly related to the PRRSV concentration in the serum [35]. However, the lack of statistical significance is likely attributable to (1) a small number of pigs per treatment group (*n* = 8–10); (2) a non-normal distribution of viral load data, which reduces statistical power, even with nonparametric tests [36]; and (3) well-documented high inter-individual variability in PRRSV viremia following experimental challenge. In PRRSV challenge studies, it is not uncommon to observe a wide range of viral titers within the same treatment group [37]. Therefore, descriptive statistics are more informative than *p*-values alone in such exploratory studies.

Importantly, the 65% reduction in serum viral load at the 0.4% D-Strike level represents a substantial decrease from a virological and clinical standpoint. A direct correlation has been established between the serum PRRSV load and severity of lung pathology. The present findings demonstrate that a lactylated fatty acid-based anionic surfactant (D-Strike) exerts similar antiviral activity in vivo. However, the lack of statistical significance for serum viral load reduction is a notable limitation of this study. While a 65% numerical reduction in viremia was observed at the 0.4% inclusion level, this finding should be interpreted as a trend rather than conclusive evidence of antiviral efficacy in vivo. It is important to emphasize the distinction between statistically supported findings and descriptive trends in this study. While the in vitro data clearly demonstrate statistically significant virus inactivation, the in vivo results—except for the body temperature and weight gain comparisons with the negative control—are primarily descriptive. The numerical reductions in viremia (65% at 0.4% D-Strike) and mortality (20% to 0%) are encouraging trends but do not reach statistical significance. These findings should be viewed as hypothesis-generating rather than confirmatory. The fact that the 0.4% inclusion rate produced a 65% reduction in serum virus load is particularly noteworthy given that some other fatty acid-based feed additives (decanoic monoglyceride and monolaurin) have failed to significantly reduce the amount of PRRSV RNA in feed or serum [22]. This suggests that the specific lactylation and surfactant properties of D-Strike may enhance its bioavailability or direct virucidal activity compared to unmodified MCFA blends.

No significant differences were detected among groups on day 11 (3 days post-challenge). This is not surprising, as this point represents the very early stage of viremia when viral replication is just beginning to peak. Most PRRSV challenge studies report peak viremia between 7 and 14 days post-infection [38]. The divergence in viral loads between treated and control groups became more apparent by the time of euthanasia (10 days post-challenge). This delayed effect suggests that D-Strike may act primarily by limiting viral dissemination or enhancing viral clearance rather than completely preventing an initial infection. Such a mechanism is plausible given that MCFAs have been shown to modulate host immune responses, including the enhancement of macrophage phagocytic activity and reduced production of pro-inflammatory cytokines [39].

An important consideration in interpreting the in vivo findings is the composition of the basal diet, which included pharmacological zinc (3000 ppm), elevated copper (199 ppm), and super-dose phytase (2000 FTU/kg). These additives are known to influence immune function and disease resistance independently of the mMCFA treatment. Pharmacological zinc has well-documented immunomodulatory effects, including enhancement of antibody responses and a reduction in inflammatory cytokine production. Elevated copper also possesses antimicrobial properties and may influence immune cell function. Phytase, while primarily added to improve phosphorus bioavailability, can influence mineral absorption and thereby indirectly affect immune function. Therefore, the observed benefits in D-Strike-fed pigs may reflect either an additive or synergistic effect with these basal diet components. Future studies should evaluate mMCFA in the context of standard commercial diets without these additional bioactive compounds to isolate the specific contribution of mMCFA to disease mitigation

PRRSV infection is well known to cause profound growth depression, driven by anorexia, fever-induced catabolism, and a diversion of nutrients toward immune responses. Schweer et al. (2016) found that the average daily gain (ADG) was reduced by ~30% and Daily Feed Intake (ADFI) by 26% over 21 days of experimental PRRSV infection compared to the controls [40]. In the present study, all PRRSV-challenged groups had significantly lower body weight on the day of challenge (Day 8) and at euthanasia (Day 18) compared to the non-challenge control (Table 5). However, when comparing the PRRSV-challenge control with D-Strike groups, no statistically significant improvements in final body weight or cumulative average daily gain (ADG) were observed (Table 5). Nonetheless, several descriptive trends merit discussion. During Phase 1 (d0–d8, before challenge), pigs assigned to D-Strike 0.2% had a numerically higher ADG (0.525 lbs) than the positive control (0.286 lbs), and this value was intermediate (superscript “ab”), suggesting that D-Strike may have conferred some pre-challenge benefits, possibly through improved gut health or reduced transport-related stress [25]. However, the D-Strike 0.4% group did not show this advantage.

During Phase 2 (d9–d10, the immediate post-challenge period), the ADG values were low in all challenged groups (0.529–0.661 lbs) and did not differ statistically. The very short duration of Phase 2 (only 2 days) may have been insufficient to capture growth differences, as PRRSV-induced anorexia typically occurs at 7–10 days post-infection [41]. A longer observation period would be needed to detect potential recovery of growth in D-Strike-treated pigs.

Cumulative ADG from d0 to euthanasia (Day 18) was 0.528 lbs and 0.489 lbs for D-Strike 0.2% and 0.4%, respectively, compared to 0.461 lbs for the PRRSV-challenge control. While not significantly different, the numerical advantage (approximately 14% higher for 0.2% D-Strike) is biologically consistent with the reduced mortality, viremia, and clinical signs. The lack of statistical significance is again likely due to small group sizes and high individual variability in feed intake and weight gain post-challenge. Notably, the feed conversion ratio (FCR) was descriptively better (lower) in the D-Strike 0.2% group (1.38) compared to the PRRSV-challenge control (1.55) and even the non-challenge control (1.64). An improved FCR suggests that pigs fed D-Strike utilized feed more efficiently despite the infection—a finding that warrants further investigation.

Additional research is needed to determine if this proprietary mMCFA can inactivate other porcine viruses or not. Piglets fed diets containing lauric and myristic acid as mMCFA had an increased average daily gain and rich microbiota but lower counts of enterotoxigenic *Escherichia* coli [42,43]. Thus, the mMCFA used in this study could be potentially useful for animals under sub-optimal conditions with overactive immune responses and cytokine storms associated with pathogenic infections, thereby reducing the serious consequences of acute infection [44].

However, several limitations of this study warrant consideration. First, the in vivo experiment used a relatively small sample size (*n* = 8–10 pigs per group), which limited statistical power to detect differences in viral load, growth performance, and other continuous outcomes. The small group size also precluded robust subgroup analyses or adjustments for confounding variables. Second, three pigs were excluded due to diarrhea upon arrival, and three additional pigs died 1–2 days before euthanasia, resulting in unequal group sizes (9, 10, 10, and 8 pigs in groups A–D, respectively). Third, the study’s duration (10 days post-challenge) may have been insufficient to capture the full growth performance outcomes. Fourth, this study was conducted in a single isolation facility with a single viral strain, limiting the generalizability of the findings. Fifth, the use of a feed additive containing other bioactive compounds (pharmacological zinc at 3000 ppm, elevated copper at 199 ppm, and super-dose phytase at 2000 FTU/kg) may have influenced the immune responses independently of D-Strike, as discussed above. Finally, the descriptive nature of several outcome measures (clinical signs, lesion severity) introduces the potential for observer bias, although efforts were made to minimize this through standardized scoring protocols and blinding.

## 5. Conclusions

The current results confirm that a proprietary blend of mMCFA can inactivate PRRSV in vitro, with the higher concentrations (1:1000 and 1:2000) eliminating at least 3 logs of PRRSV within 5 min. The dietary D-Strike showed favorable numerical trends in vivo by reducing clinical signs, as well as febrile response, lung lesion severity, and mortality. These clinical benefits are mechanistically linked to the reduced serum viremia and lung lesion severity. However, the lack of statistical significance for viremia reduction, combined with the small sample size and high inter-individual variability, precludes definitive conclusions about in vivo antiviral efficacy. The apparent dose-dependent trends observed for several outcomes (mortality, lung lesion severity, and viral load) show the promise of D-Strike but require confirmation in larger, appropriately powered studies. The mMCFA shows promise as a feed additive that could potentially be used during outbreaks as a supportive substance to minimize the severity and losses related to PRRSV infection.

## Figures and Tables

**Table 1:** Groups of pigs and treatments.

Group No.	Treatment	Amount of D-Strike Added to Feed	Virus Challenge
A	Negative control	0	No
B	Positive control	0	Yes
C	Low dose of mMCFA	4 lbs/ton of feed	Yes
D	High dose of mMCFA	8 lbs/ton of feed	Yes

**Table 2:** Chronology of treatments given to pigs.

Days	Task
1–7	Arrival of pigs; random assignment to 4 groups; test for PRRSV by rRT-PCR
8	Groups B, C, and D challenged with PRRSV
9–17	Monitoring of clinical signs and temperature
11	Blood collection to test for PRRSV in serum
18	Blood collection, euthanasia, and necropsy

**Table 3:** In vitro inactivation of PRRSV by mMCFA.

Initial Dilution of mMCFA	Final Dilution After Mixing with an Equal Amount of Virus	Mean ± SD of Virus Titer as Log10 TCID50/0.1 mL After the Indicated Amount of Time (Percentage of Virus Inactivation)		
		1 min	2 min	5 min
1:1000	1:2000	1.16 ± 0.34 (99.95)	0.83 ± 0.00 (99.97)	<1.0 ± 0.00 (>99.99)
1:2000	1:4000	1.83 ± 0.33 (99.78)	0.83 ± 0.00 (99.97)	<1.0 ± 0.00 (>99.99)
1:5000	1:10,000	1.83 ± 0.33 (99.78)	0.83 ± 0.00 (99.97)	<1.0 ± 0.00 (>99.99)
1:10,000	1:20,000	4.49 ± 0.58 (0.00)	4.50 ± 0.00 (0.00)	4.16 ± 0.00 (53.00)

Note 1: The initial virus titer was 4.5 log10 TCID_50_/0.1 mL. Note 2: Percent virus inactivation was calculated by the formula (A − B/A) × 100, where A = initial virus titer and B = remaining virus titer after interaction with mMCFA in a suspension test.

**Table 4:** Mortality and the severity of histopathological lesions in the lungs of pigs.

Group No.	Treatment	No. of Pigs	Percent *	No. (%) of Pigs Showing the Indicated Lesions			
				None	Mild	Moderate	Severe
A	Negative control	9	0	9 (100)	0 (0)	0 (0)	0 (0)
B	Positive control	10	20	0 (0)	0 (0)	1 (12.5)	7 (87.5)
C	Low dose of mMCFA	10	10	0 (0)	1 (11.1)	1 (11.1)	7 (77.8)
D	High dose of mMCFA	8	0	0 (0)	1 (12.5)	3 (37.5)	4 (50)

* Percentage of mortality after PRRSV challenge.

**Table 5:** Pigs’ average performance and body weight gain.

Groups	A	B	C	D	SE
	Non-Challenge Control	PRRSV-Challenge Control	D-Strike 0.2%	D-Strike 0.4%	
Initial BW, lbs	12.959	13.204	13.184	13.366	0.64
Day of challenge (Day 8)	19.213	15.499	17.395	15.549	0.86
Day of euthanasia (Day 18)	32.767 a	21.5 b	22.68 b	22.175 b	1.29
Phase 1 d0–d8	0.782 a	0.286 b	0.525 ab	0.275 b	0.06
Phase 2 d9–10	1.354 a	0.600 b	0.529 b	0.661 b	0.09
ADG, lbs	1.102 a	0.461 b	0.528 b	0.489 b	0.05
Total Weight Gain	19.808 a	8.296 b	9.496 b	8.809 b	0.89
FCR	1.64	1.55	1.38	1.54	NA

BW, body weight; SE, standard error; ADG, average daily gain; FCR, feed conversion ratio; NA, not applicable. Note 1. Groups with different letters (e.g., 32.767 a vs. 21.5 b) are significantly different from each other. Note 2. Groups sharing a letter (e.g., 22.68 b and 22.175 b) are not significantly different.

## Data Availability

The original contributions presented in this study are included in the article. Further inquiries can be directed to the corresponding authors.

## Abbreviations

The following abbreviations are used in this manuscript:

PRRSV	Porcine reproductive and respiratory syndrome virus
MCFA	Medium-chain fatty acids
mMCFA	Modified medium-chain fatty acids
V-qPCR	Viability qPCR
MEM	Modified Eagle’s medium
IACUC	Institutional Animal Care and Use Committee
ANOVA	Analysis of Variance
qRT-PCR	Quantitative Reverse Transcription Polymerase Chain Reaction
ADFI	Daily Feed Intake
ADG	Average daily gain
FCR	Feed conversion ratio

## References

[1] Nathues H., Alarcon P., Rushton J., Jolie R., Fiebig K., Jimenez M., Geurts V., Nathues C. (2017). Cost of porcine reproductive and respiratory syndrome virus at individual farm level–An economic disease model. Prev. Vet. Med..

[2] Chen X.X., Qiao S., Li R., Wang J., Li X., Zhang G. (2023). Evasion strategies of porcine reproductive and respiratory syndrome virus. Front. Microbiol..

[3] Zhang Z., Zhang H., Liu T. (2019). Study on body temperature detection of pig based on infrared technology: A review. Artif. Intell. Agric..

[4] Chase-Topping M., Xie J., Pooley C., Trus I., Bonckaert C., Rediger K., Bailey R.I., Brown H., Bitsouni V., Barrio M.B. (2020). New insights about vaccine effectiveness: Impact of attenuated PRRS-strain vaccination on heterologous strain transmission. Vaccine.

[5] Wills R.W., Doster A.R., Galeota J.A., Sur J.H., Osorio F.A. (2003). Duration of infection and proportion of pigs persistently infected with porcine reproductive and respiratory syndrome virus. J. Clin. Microbiol..

[6] Zhao D., Yang B., Yuan X., Shen C., Zhang D., Shi X., Zhang T., Cui H., Yang J., Chen X. (2021). Advanced research in porcine reproductive and respiratory syndrome virus co-infection with other pathogens in swine. Front. Vet. Sci..

[7] Jadhav H.B., Annapure U.S. (2022). Triglycerides of medium-chain fatty acids: A concise review. J. Food Sci. Technol..

[8] Burdock G.A., Carabin I.G. (2007). Safety assessment of myristic acid as a food ingredient. Food Chem. Toxicol..

[9] Zeng X., Li S., Ye Q., Cai S., Quan S., Liu L., Zhang S., Chen F., Cai C., Wang F. (2022). The combined use of medium-and short-chain fatty acids improves the pregnancy outcomes of sows by enhancing ovarian steroidogenesis and endometrial receptivity. Nutrients.

[10] Correa-Fiz F., Neila-Ibáñez C., López-Soria S., Napp S., Martinez B., Sobrevia L., Tibble S., Aragon V., Migura-Garcia L. (2020). Feed additives for the control of post-weaning *Streptococcus suis* disease and the effect on the faecal and nasal microbiota. Sci. Rep..

[11] Baltić B., Starčević M., Đorđević J., Mrdović B., Marković R. (2017). Importance of medium-chain fatty acids in animal nutrition. IOP Conf. Ser. Earth Environ. Sci..

[12] Lerner A.B., Cochrane R.A., Gebhardt J.T., Dritz S.S., Jones C.K., DeRouchey J.M., Tokach M.D., Goodband R.D., Bai J., Porter E. (2020). Effects of medium-chain fatty acids as a mitigation or prevention strategy against porcine epidemic diarrhea virus in swine feed. J. Anim. Sci..

[13] Jackman J.A., Hakobyan A., Zakaryan H., Elrod C.C. (2020). Inhibition of African swine fever virus in liquid and feed by medium-chain fatty acids and glycerol monolaurate. Anim. Sci. Biotechnol..

[14] Dee S.A., Niederwerder M.C., Edler R., Hanson D., Singrey A., Cochrane R., Spronk G., Nelson E. (2021). An evaluation of additives for mitigating the risk of virus-contaminated feed using an ice-block challenge model. Transbound. Emerg. Dis..

[15] Su B., Wang Y., Jian S., Tang H., Deng H., Zhu L., Zhao X., Liu J., Cheng H., Zhang L. (2023). In vitro and in vivo antiviral activity of monolaurin against Seneca Valley virus. Front. Vet. Sci..

[16] Kim H.S., Kwang J., Yoon I.J., Joo H.S., Frey M.L. (1993). Enhanced replication of porcine reproductive and respiratory syndrome (PRRS) virus in a homogeneous subpopulation of MA-104 cell line. Arch. Virol..

[17] Balestreri C., Schroeder D.C., Sampedro F., Marqués G., Palowski A., Urriola P.E., van de Ligt J.L., Yancy H.F., Shurson G.C. (2024). Unexpected thermal stability of two enveloped megaviruses, Emiliania huxleyi virus and African swine fever virus, as measured by viability PCR. Virol. J..

[18] Quinonez-Munoz A., Sobhy N.M., Goyal S.M. (2024). Comparative survival of five porcine reproductive and respiratory syndrome virus strains on six fomites. Vet. World.

[19] Zentek J., Buchheit-Renko S., Ferrara F., Vahjen W., Van Kessel A.G., Pieper R. (2011). Nutritional and physiological role of medium-chain triglycerides and medium-chain fatty acids in piglets. Anim. Health Res. Rev..

[20] Slaoui M., Fiette L. (2011). Histopathology procedures: From tissue sampling to histopathological evaluation. Drug Safety Evaluation; Methods in Molecular Biology.

[21] Valle-González E.R., Jackman J.A., Yoon B.K., Park S., Sut T.N., Cho N.J. (2018). Characterizing how acidic pH conditions affect the membrane-disruptive activities of lauric acid and glycerol monolaurate. Langmuir.

[22] Yang L., Wen J., Zhang Y., Liu Z., Luo Z., Xu L., Lai S., Tang H., Sun X., Hu Y. (2022). The antiviral activity of caprylic monoglyceride against porcine reproductive and respiratory syndrome virus in vitro and in vivo. Molecules.

[23] Tran H.T.T., Truong A.D., Ly D.V., Van Hoang T., Chu N.T., Nguyen H.T., Dang A.T.K., De Vos M., Lannoo K., Bruggeman G. (2021). The potential anti-African swine fever virus effects of medium chain fatty acids on in vitro feed model: An evaluation study using epidemic ASFV strain circulating in Vietnam. Open Vet. J..

[24] Hornung B., Amtmann E., Sauer G. (1994). Lauric acid inhibits the maturation of vesicular stomatitis virus. J. Gen. Virol..

[25] Jackman J.A., Boyd R.D., Elrod C.C. (2020). Medium-chain fatty acids and monoglycerides as feed additives for pig production: Towards gut health improvement and feed pathogen mitigation. J. Anim. Sci. Biotechnol..

[26] Dayrit F.M. (2015). The properties of lauric acid and their significance in coconut oil. J. Am. Oil Chem. Soc..

[27] Dierick N.A., Decuypere J.A., Molly K., Van B.E., Vanderbeke E. (2002). The combined use of triacylglycerols (TAGs) containing medium chain fatty acids (MCFAs) and exogenous lipolytic enzymes as an alternative to nutritional antibiotics in piglet nutrition: II. In vivo release of MCFAs in gastric cannulated and slaughtered piglets by endogenous and exogenous lipases; effects on the luminal gut flora and growth performance. Livest. Prod. Sci..

[28] Majumder S., Saha M. (2025). Porcine reproductive and respiratory syndrome (PRRS): A comprehensive review of recent developments in diagnosis and control strategies. J. Adv. Biol. Biotechnol..

[29] Rimayanti R., Khairullah A.R., Lestari T.D., Hernawati T., Mulyati S., Utama S., Damayanti R., Moses I.B., Yanestria S.M., Kusala M.K.J. (2024). Porcine reproductive and respiratory syndrome developments: An in-depth review of recent findings. Open Vet. J..

[30] Ferlazzo G., Ruggeri J., Boniotti M.B., Guarneri F., Barbieri I., Tonni M., Bertasio C., Alborali G.L., Amadori M. (2020). In vitro cytokine responses to virulent PRRS virus strains. Front. Vet. Sci..

[31] Soerensen D.D., Pedersen L.J. (2015). Infrared skin temperature measurements for monitoring health in pigs: A review. Acta Vet. Scand..

[32] Schul W., Liu W., Xu H.Y., Flamand M., Vasudevan S.G. (2007). A dengue fever viremia model in mice shows reduction in viral replication and suppression of the inflammatory response after treatment with antiviral drugs. J. Infect. Dis..

[33] Risley C., Lopez J., Cordts C. (2024). PSV-18 improved performance of PRRSV challenged pigs fed 1-α-glycerol monolaurate. J. Anim. Sci..

[34] Lim B., Kim S.C., Kim H.J., Kim J.H., Seo Y.J., Lim C., Park Y., Sheet S., Kim D., Lim D.H. (2025). Single-cell transcriptomics of bronchoalveolar lavage during PRRSV infection with different virulence. Nat. Commun..

[35] Loving C.L., Brockmeier S.L., Vincent A.L., Lager K.M., Sacco R.E. (2008). Differences in clinical disease and immune response of pigs challenged with a high-dose versus low-dose inoculum of porcine reproductive and respiratory syndrome virus. Viral Immunol..

[36] Pasternak J.A., MacPhee D.J., Harding J.C. (2020). Maternal and fetal thyroid dysfunction following porcine reproductive and respiratory syndrome virus2 infection. Vet. Res..

[37] Raev S.A., Cai L., Muro N., Madera R., Wang L., Shi J. (2026). Cross-protection in PRRSV: Mechanisms, limitations, and implications for vaccine design. Pathogens.

[38] Duerlinger S., Knecht C., Sawyer S., Balka G., Zaruba M., Ruemenapf T., Kraft C., Rathkjen P.H., Ladinig A. (2022). Efficacy of a modified live porcine reproductive and respiratory syndrome virus 1 (PRRSV-1) vaccine against experimental infection with PRRSV AUT15-33 in weaned piglets. Vaccines.

[39] Kim C.H. (2026). Short-, medium-, versus long-chain fatty acids: Mechanisms of immunomodulation and disease pathogenesis. Cell. Mol. Immunol..

[40] Schweer W.P., Schwartz K., Burrough E.R., Yoon K.J., Sparks J.C., Gabler N.K. (2016). The effect of porcine reproductive and respiratory syndrome virus and porcine epidemic diarrhea virus challenge on growing pigs I: Growth performance and digestibility. J. Anim. Sci..

[41] Tong G.Z., Zhou Y.J., Hao X.F., Tian Z.J., An T.Q., Qiu H.J. (2007). Highly pathogenic porcine reproductive and respiratory syndrome, China. Emerg. Infect. Dis..

[42] Bai G., He W., Yang Z., Fu H., Qiu S., Gao F., Shi B. (2019). Effects of different emulsifiers on growth performance, nutrient digestibility, and digestive enzyme activity in weanling pigs. J. Anim. Sci..

[43] Wang X., Tsai T., Wei X., Zuo B., Davis E., Rehberger T., Hernandez S., Jochems E.J., Maxwell C.V., Zhao J. (2021). Effect of Lactylate and Bacillus subtilis on growth performance, peripheral blood cell profile, and gut microbiota of nursery pigs. Microorganisms.

[44] Fosdick M.G., Chheda P.R., Tran P.M., Wolff A., Peralta R., Zhang M.Y., Kerns R., Houtman J.C. (2021). Suppression of human T cell activation by derivatives of glycerol monolaurate. Sci. Rep..

